# Causal effects from inflammatory bowel disease on liver function and disease: a two-sample Mendelian randomization study

**DOI:** 10.3389/fmed.2023.1320842

**Published:** 2024-01-17

**Authors:** Yufeng Shu, Bocheng Yang, Xuanyou Liu, Meihua Xu, Chao Deng, Hao Wu

**Affiliations:** ^1^Department of Gastroenterology, Third Xiangya Hospital, Central South University, Changsha, China; ^2^Division of Plastic Surgery, Zhongshan Hospital Xiamen University, Xiamen, China; ^3^Department of Molecular Medicine, Mayo Clinic, Rochester, MN, United States; ^4^Department of Gastroenterology, Xiangya Hospital, Central South University, Changsha, China; ^5^Department of Orthopaedics, Xiangya Hospital, Central South University, Changsha, China

**Keywords:** ulcerative colitis, Crohn’s disease, non-alcoholic fatty liver disease, primary sclerosing cholangitis, primary biliary cholangitis

## Abstract

**Background:**

Accumulating evidence has shown that patients with inflammatory bowel disease (IBD) have liver function abnormalities and are susceptible to liver diseases. However, the existence of a causal relationship between IBD and liver function or disease remains unclear.

**Methods:**

A two-sample Mendelian randomization (MR) analysis was performed using genetic associations from publicly available genome-wide association studies (GWAS). These associations encompass ulcerative colitis (UC), Crohn’s disease (CD), liver function traits, and liver disease phenotypes. The liver function traits comprised hepatic biochemistries, percent liver fat, and liver iron content from the UK Biobank. Furthermore, the liver disease phenotypes included cholelithiasis, non-alcoholic fatty liver disease (NAFLD), primary sclerosing cholangitis (PSC), and primary biliary cholangitis (PBC) in cohorts of European ancestry. The primary estimation used the inverse-variance weighted method, with GWAS of C-reactive protein (CRP) in the UK Biobank serving as a positive control outcome.

**Results:**

Genetically predicted UC is causally associated with decreased levels of albumin (ALB) and liver iron content, while genetically predicted CD is causally associated with increased levels of alkaline phosphatase (ALP). Moreover, genetically predicted UC or CD increases the risk of PSC, and CD increases the risk of PBC. Neither UC nor CD causally increases the risk of cholelithiasis and NAFLD.

**Conclusion:**

UC affects the levels of ALB and liver iron content, while CD affects the levels of ALP. Both UC and CD increase the risk of PSC, and CD increases the risk of PBC.

## 1 Introduction

Inflammatory bowel disease (IBD), which comprises ulcerative colitis (UC) and Crohn’s disease (CD), is a global disease that affects people from diverse regions and ethnic backgrounds worldwide (1). IBD is a systemic disease associated with notable extraintestinal manifestations, including but not limited to cardiovascular disease, hepatobiliary disease, arthropathy, arthritis, and skin disease (2). The prevalence of abnormal hepatic biochemistries in IBD patients ranges from 3 to 50% in different studies, and approximately 5% of IBD patients will develop liver disease (3).

The most common liver disease associated with IBD is non-alcoholic fatty liver disease (NAFLD), whereas the most specific liver disease associated with IBD is primary sclerosing cholangitis (PSC). A meta-analysis revealed that the pooled prevalence of non-alcoholic fatty liver disease (NAFLD) was 30.7% in patients with IBD, and IBD patients had a 2-fold higher risk of developing NAFLD compared to healthy individuals (4). The pooled prevalence of PSC in IBD was 2.16%, whereas up to 85% of patients with PSC suffer from IBD (5, 6). In addition, a case–control study found that patients with CD but not UC have a significantly higher risk of developing cholelithiasis than well-matched hospital controls (7). However, these observational studies inherently have limitations in terms of confounding bias or reverse causation, and the existence of a causal relationship between IBD and liver function or disease remains unclear.

Mendelian randomization (MR) is a more reliable approach to assess causal relationships using observational data. MR is a technique that leverages genetic variants to make inferences about the causal effect of an exposure on an outcome (8). Results from MR have often been shown to qualitatively agree with the results from randomized trials. A two-sample MR analysis was carried out in this study to evaluate the causal effects of IBD on liver function and disease using available large-scale genome-wide association study (GWAS) data. The liver function traits include hepatic biochemistries, percent liver fat, and liver iron content in the UK Biobank. Additionally, the liver disease phenotypes include cholelithiasis, NAFLD, PSC, and primary biliary cholangitis (PBC) in cohorts of European ancestry. Indeed, the latest MR study revealed that genetically predicted IBD can increase the risk of PBC in the European population (9). We aimed to provide a comprehensive assessment of the causal role of IBD in relation to liver health and disease.

## 2 Methods

### 2.1 GWAS data sources for inflammatory bowel disease

The International Inflammatory Bowel Disease Genetics Consortium (IIBDGC, https://www.ibdgenetics.org/) provided the available GWAS datasets for IBD, including UC (dataset: ieu-a-970) and CD (dataset: ieu-a-12). IIBDGC comprised 13,768 cases of UC and 33,977 controls, as well as 17,897 cases of CD and 33,977 controls, all individuals of European ancestry. The diagnosis of IBD was determined through accepted radiologic, endoscopic, and histopathologic evaluations (10).

### 2.2 GWAS data sources for liver function traits and liver disease phenotypes

We utilized summary-level data from published GWASs of liver function traits in the UK Biobank, with a participant range of 273,896 to 344,292 individuals of European ancestry. The GWASs focused on various liver function traits, including albumin (ALB), aspartate aminotransferase (AST), alanine transaminase (ALT), alkaline phosphatase (ALP), gamma-glutamyl transferase (γ-GGT), direct bilirubin (DBIL), total bilirubin (TBIL), lipoprotein (a) (Lp(a)), total cholesterol (TC), triglyceride (TG), low-density lipoprotein (LDL), high-density lipoprotein (HDL), apolipoprotein A1 (ApoA1), and apolipoprotein B (ApoB). Based on the information from the UK Biobank, ALB levels were assessed using bromocresol green analysis, while AST, ALT, ALP, and γ-GGT levels were determined through the International Federation of Clinical Chemistry and Laboratory Medicine (IFCC) analysis. Direct bilirubin levels were measured using the diazotization-spectrophotometric method, and total bilirubin levels were determined through photometric color analysis. HDL levels were analyzed using enzyme immunoinhibition and ApoA1 and ApoB levels through immunoturbidimetric analysis. TG levels were measured using the glycerol-3-phosphate oxidase-peroxidase method, while LDL levels were determined via enzymatic protective selection analysis. TC levels were established using the cholesterol oxidase-peroxidase (CHO-POD) enzymatic method. All these biomarkers were assessed using a Beckman Coulter AU5800. Furthermore, Lp(a) levels were measured through immunoturbidimetric analysis using a Randox AU5800. The summary-level GWAS data on percent liver fat and liver iron content were collected from a published article, which involved over 38,000 abdominal MRI scans conducted in the UK Biobank (11). The MRI measurements are performed using a Siemens 1.5 T MAGNETOM Aera. Derived measures of liver fat and liver iron content were generated by Perspectum Diagnostics (https://www.perspectum.com/). The summary-level GWAS data for NAFLD were acquired from a meta-analysis conducted on the data from the Electronic Medical Records and Genomics (eMERGE) network, the UK Biobank, the Estonian Biobank, and FinnGen, as documented in the GWAS Catalog (www.ebi.ac.uk/gwas/studies/GCST90091033). The meta-analysis of GWAS data for NAFLD included 8,434 cases of European ancestry and 770,180 European ancestry controls (12). The diagnosis of NAFLD within the eMERGE network utilized ICD-9 and ICD-10 codes (ICD-9: 571.5, 571.8, 571.9; ICD-10: K75.81, K76.0, K76.9), while the UK Biobank and the Estonian Biobank employed ICD-10 codes (ICD-10: K74.0, K74.2, K75.8, K76.0, and K76.9). In FinnGen, NAFLD was defined based on ICD-10 code K76.0. As suggested by the American Association for the Study of Liver Disease (AASLD), the study excluded secondary causes (alcohol dependence, hepatitis, liver transplant, cystic fibrosis, etc.) of NAFLD whenever possible. The summary-level GWAS data related to cholelithiasis were acquired from the FinnGen consortium, Beta 8 (32,894 European ancestry cases and 301,383 European ancestry controls). The definition of cholelithiasis was based on ICD-10 code K80. The summary-level GWAS data for PBC were acquired from a meta-analysis conducted on the data from five cohorts of European ancestry (8,021 cases and 16,489 controls, www.ebi.ac.uk/gwas/studies/GCST90061440). All the PBC cases were defined by the criteria of the European Association for the Study of the Liver (EASL) (13). The summary-level GWAS data for PSC were collected from Scandinavia, Germany, the UK, and the US cohorts (2,871 cases and 12,019 controls, www.ebi.ac.uk/gwas/studies/GCST004030) (14). PSC diagnosis relied on standard clinical, biochemical, cholangiographic, and histological criteria while excluding secondary causes of sclerosing cholangitis (15).

### 2.3 Positive control and replication analysis

C-reactive protein (CRP) is well-established as both a predictive factor and a marker of inflammation in IBD. In addition, a prior Mendelian randomization study demonstrated that increased levels of CRP exposure did not elevate the risk of developing inflammatory bowel disease (16). To validate our study design, we incorporated summary-level data from published GWAS of CRP in UK Biobank (N = 343,524 European ancestry individuals) as a positive control outcome in our study. The CRP levels were measured by high-sensitivity immunoturbidimetry analysis on a Beckman Coulter AU5800. In the replication analysis, a combined meta-analysis of the UK Inflammatory Bowel Disease Genetics Consortium (UKIBDGC) and the IIBDGC included 12,366 UC cases and 33,609 controls, along with 12,194 CD cases and 28,072 controls. The diagnosis of IBD was also determined through accepted radiologic, endoscopic, and histopathologic evaluations. The GWAS data were downloaded from the Wellcome Sanger Institute database (https://ftp.sanger.ac.uk/pub/project/humgen/summary_statistics/human/2016-11-07/) (17).

### 2.4 Genetic instrumental variable selection

The criteria for genetic instrumental variables (IVs) selection were introduced in our previous study (18). In order to test the presence of weak instrumental variable bias, we calculated *F* statistics using the formula *F = R^2^(n-k-1*)*/k(1-R^2^)*, where *R^2^* is the proportion of the variance of the exposure explained by the IVs, *n* sample size, and *k* number of genetic variants. If the *F* statistics for the instrument-exposure association exceed 10, the likelihood of weak instrumental variable bias is minimal. From the European ancestry participants of the IIBDGC, we identified 86 IVs associated with UC and 115 IVs associated with CD. Similarly, from the European ancestry participants within the combined UKIBDGC and IIBDGC, we identified 62 index SNPs as instrumental variables for UC, as well as 89 index SNPs for CD. Before each MR analysis, we applied the MR Pleiotropy RESidual Sum and Outlier (MR-PRESSO) method to identify and remove any potential outliers.

### 2.5 Mendelian randomization estimates

The random-effect inverse-variance weighted (IVW) method, known for its efficiency in incorporating valid IVs, was used as the primary analysis (19). MR-Egger and weighted median (WM) methods were used as robust methods for sensitivity analysis. When the estimates of direction derived from IVW, MR-Egger, and WM methods are consistent, it enhances the credibility of the causal claim (20, 21). Cochran’s Q statistic was utilized to assess heterogeneity between variant-specific causal estimates. To assess horizontal pleiotropy, the Egger intercept test and leave-one-out (LOO) analyses were employed. In addition, the Steiger test was used to validate the direction of observed causalities (22). All MR analyses were performed using the R package two Sample MR (version 0.5.6) and MR-PRESSO (version 1.0). A *p*-value of <0.05 was considered statistically significant.

## 3 Results

**Figure 1:**
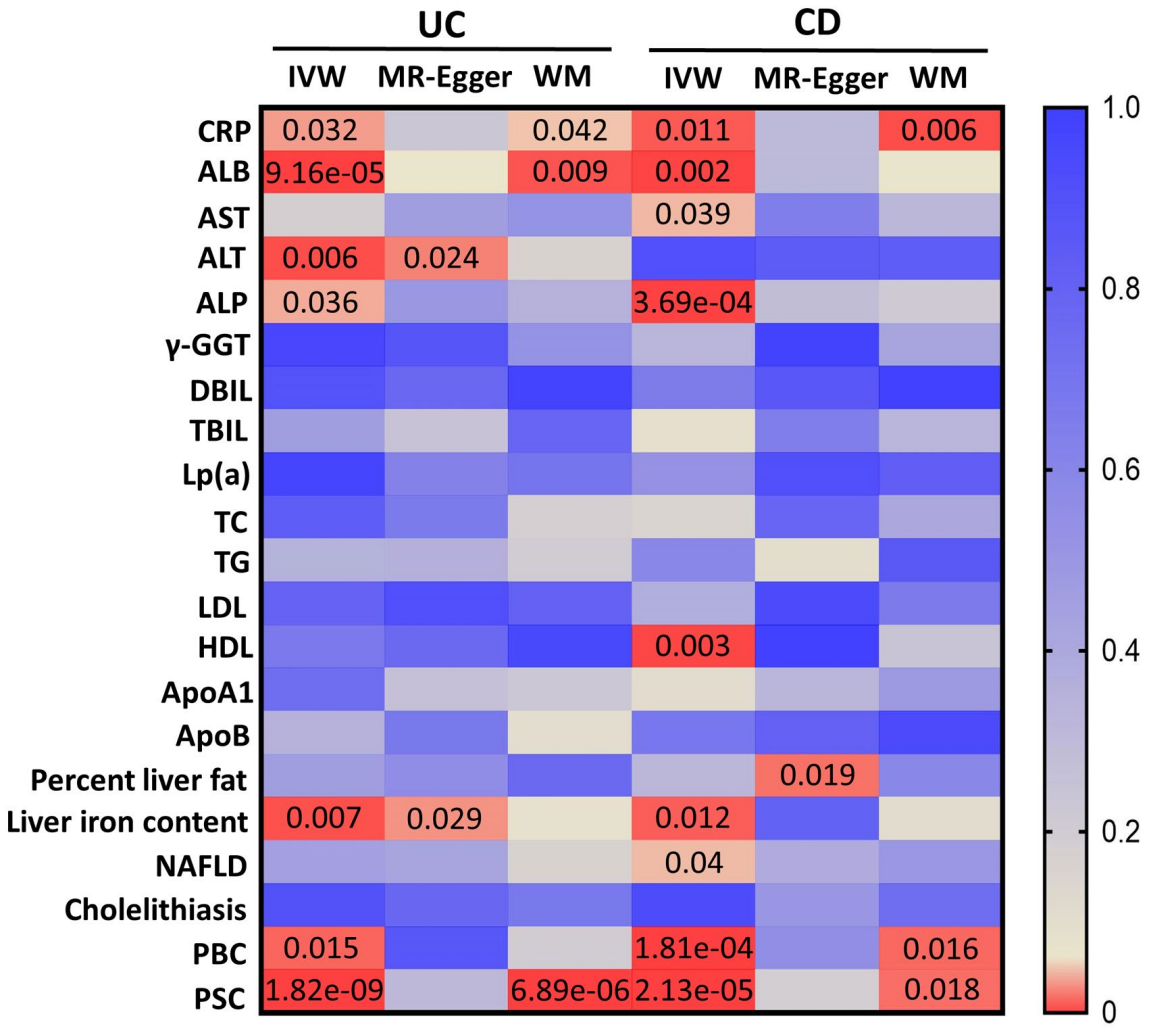
MR estimates from UC and CD on liver function traits and liver disease phenotypes. The color of each block represents the *p*-values of every MR analysis. CRP, C-reactive protein; ALB, albumin; AST, aspartate aminotransferase; ALT, alanine transaminase; ALP, alkaline phosphatase; γ-GGT, gamma-glutamyl transferase; DBIL, direct bilirubin; TBIL, total bilirubin; Lp(a), lipoprotein (a); TC, total cholesterol; TG, triglyceride; LDL, low-density lipoprotein; HDL, high-density lipoprotein; ApoA1, apolipoprotein A1; ApoB, apolipoprotein B; NAFLD, non-alcoholic fatty liver disease; PBC, primary biliary cholangitis; PSC, primary sclerosing cholangitis; UC, ulcerative colitis; CD, Crohn’s disease; IVW, inverse-variance weighted; WM, weighted median. A *p*-value of <0.05 is set as significant.

Details of all GWASs included in our study are presented in Table 1. In total, 86 IVs (*F* = 77.48) were chosen to genetically predict UC, and 115 IVs (*F* = 89.83) were chosen to genetically predict CD. After removing all the outliers by MR-PRESSO, the MR estimates from specific methods of the causal effect of UC and CD on liver function traits and liver disease phenotypes are presented in a heatmap (Figure 1).

**Table 1:** Details of the GWASs included in the Mendelian randomization.

Trait/Phenotype	Resources	Participants
Ulcerative colitis	IIBDGC	13,768 European ancestry cases 33,977 European ancestry controls
	UKIBDGC and IIBDGC	12,366 European ancestry cases 33,609 European ancestry controls
Crohn’s disease	IIBDGC	17,897 European ancestry cases 33,977 European ancestry controls
	UKIBDGC and IIBDGC	12,194 European ancestry cases 28,072 European ancestry controls
CRP, ALB, AST, ALT, ALP, γ-GGT, DBIL, TBIL, Lp(a), TC, TG, LDL, HDL, ApoA1, ApoB	UK biobank	Range from 273,896 to 344,292 European ancestry individuals
Percent liver fat	PMID: 34128465	32,858 European ancestry individuals
Liver iron content	PMID: 34128465	32,858 European ancestry individuals
Non-alcoholic fatty liver disease	PMID: 34841290	8,434 European ancestry cases 770,180 European ancestry controls
Cholelithiasis	FinnGen consortium (Beta 8)	32,894 European ancestry cases 301,383 European ancestry controls
Primary biliary cholangitis	PMID: 34033851	8,021 European ancestry cases 16,489 European ancestry controls
Primary sclerosing cholangitis	PMID: 27992413	2,871 European ancestry cases 12,019 European ancestry controls

CRP, C-reactive protein; ALB, albumin; AST, aspartate aminotransferase; ALT, alanine transaminase; ALP, alkaline phosphatase; γ-GGT, gamma-glutamyl transferase; DBIL, direct bilirubin; TBIL, total bilirubin; Lp(a), lipoprotein (a); TC, total cholesterol; TG, triglyceride; LDL, low-density lipoprotein; HDL, high-density lipoprotein; ApoA1, apolipoprotein A1; ApoB, apolipoprotein B.

**Figure 2:**
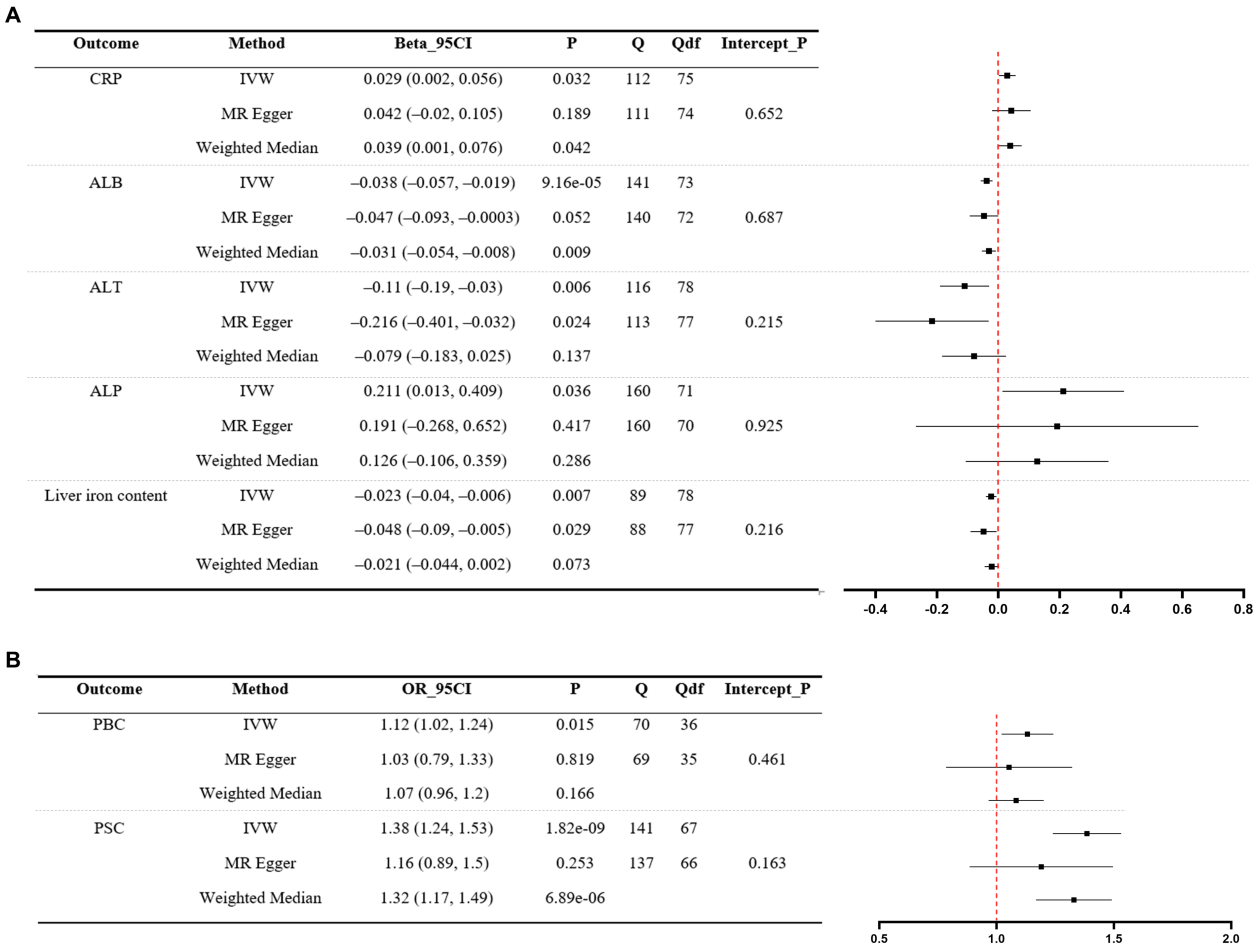
MR analyses of UC (exposure) on liver function traits (outcome) and two liver disease phenotypes (outcome). **(A)** Liver function traits. **(B)** Liver disease phenotypes. A forest plot of three estimates for each outcome is shown (IVW, MR Egger, and weighted median), along with Cochran’s Q statistic (Q) and the associated df (Qdf), and the value of p for the MR Egger intercept (Intercept_P). CRP, C-reactive protein; ALB, albumin; ALT, alanine transaminase; ALP, alkaline phosphatase; PBC, primary biliary cholangitis; PSC, primary sclerosing cholangitis; UC, ulcerative colitis; IVW, inverse-variance weighted.

Significant MR analyses of UC on liver function traits and two liver disease phenotypes are presented in Figure 2. For the positive control, the IVW indicated that genetically predicted UC was found to increase the levels of CRP (*β* = 0.029, 95%CI 0.002 to 0.056, *p* = 0.032). Among liver function traits (Figure 2A), there was evidence for an effect of UC on ALB (*β* = −0.038, 95%CI –0.057 to −0.019, *p* = 9.16e-05), ALT (*β* = −0.11, 95%CI –0.19 to −0.03, *p* = 0.006), ALP (*β* = 0.211, 95%CI 0.013 to 0.409, *p* = 0.036), and liver iron content (*β* = −0.023, 95%CI –0.04 to −0.006, *p* = 0.007). Among the liver disease phenotypes (Figure 2B), the findings demonstrated that the genetically predicted UC is causally associated with an increased risk of PBC (OR = 1.12, 95%CI 1.02 to 1.24, *p* = 0.015) and PSC (OR = 1.38, 95%CI 1.24 to 1.53, *p* = 1.82e-09). Scatter plots of significant MR estimates are shown in Supplementary Figure S1. Nevertheless, significant heterogeneity of SNP-specific causal estimates was observed, as indicated by the substantial values of Cochran’s Q statistic. In the MR-Egger intercept tests, there was no significant evidence of horizontal pleiotropy. The LOO analyses revealed the absence of potentially influential instrumental variables in UC on ALB, ALT, liver iron content, PBC, and PSC, but their presence in UC on ALP (Supplementary Figure S2). The Steiger test validated the directionality of the causal effects of UC on liver function traits and two liver disease phenotypes (Supplementary Table S1). In summary, the study yields robust conclusions that genetically predicted UC is causally associated with decreased levels of ALB, ALT, and liver iron content. Additionally, genetically predicted UC is associated with an increased risk of PBC and PSC.

**Figure 3:**
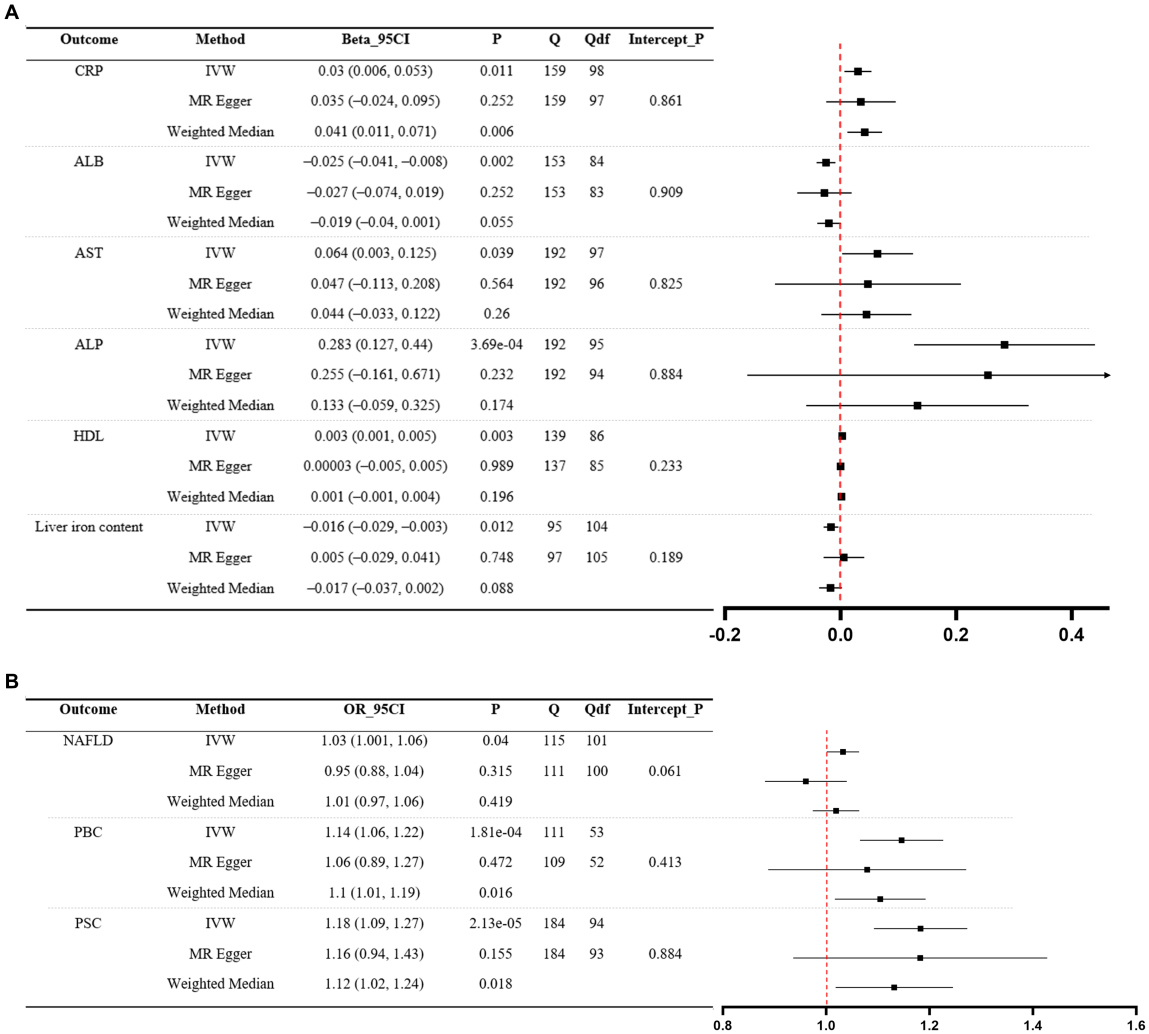
MR analyses of CD (exposure) on liver function traits (outcome) and three liver disease phenotypes (outcome). **(A)** Liver function traits. **(B)** Liver disease phenotypes. A forest plot of three estimates for each outcome is shown (IVW, MR Egger, and weighted median), along with Cochran’s Q statistic (Q) and the associated df (Qdf), and the value of p for the MR Egger intercept (Intercept_P). CRP, C-reactive protein; ALB, albumin; AST: aspartate aminotransferase; ALP, alkaline phosphatase; HDL, high-density lipoprotein; NAFLD, non-alcoholic fatty liver disease; PBC, primary biliary cholangitis; PSC, primary sclerosing cholangitis; CD, Crohn’s disease; IVW, inverse-variance weighted.

Significant MR analyses of CD on liver function traits and three liver disease phenotypes are presented in Figure 3. For the positive control, the IVW revealed that genetically predicted CD was causally associated with elevated levels of CRP (*β* = 0.03, 95%CI 0.006 to 0.053, *p* = 0.011). Among liver function traits (Figure 3A), there was evidence for an effect of CD on ALB (*β* = −0.025, 95%CI –0.041 to −0.008, *p* = 0.002), AST (*β* = 0.064, 95%CI 0.003 to 0.125, *p* = 0.039), ALP (*β* = 0.283, 95%CI 0.127 to 0.44, *p* = 3.69e-04), HDL (*β* = 0.003, 95%CI 0.001 to 0.005, *p* = 0.003), and liver iron content (*β* = −0.016, 95%CI –0.029 to −0.003, *p* = 0.012). Among the liver disease phenotypes (Figure 3B), the findings demonstrated that the genetically predicted CD was causally associated with an increased risk of NAFLD (OR = 1.03, 95%CI 1.001 to 1.06, *p* = 0.04), PBC (OR = 1.14, 95%CI 1.06 to 1.22, *p* = 1.81e-04, and PSC (OR = 1.18, 95%CI 1.09 to 1.27, *p* = 2.13e-05). Scatter plots illustrating significant MR estimates are presented in Supplementary Figure S3. Of note, the scatter plot depicting the relationship between genetically predicted CD and liver iron content, as well as NAFLD, exhibited inconsistent directional estimates from the IVW, MR-Egger, and WM methods. In addition, significant heterogeneity of SNP-specific causal estimates was observed, as indicated by the substantial values of Cochran’s Q statistic. In MR-Egger intercept tests, we detected no significant evidence of horizontal pleiotropy. The LOO analyses revealed the absence of potentially influential instrumental variables in CD on ALB, ALP, liver iron content, PBC, and PSC, but their presence in CD on AST, HDL, and NAFLD (Supplementary Figure S4). The Steiger test validated the directionality of the causal effects of CD on liver function traits and three liver disease phenotypes, but not vice versa (Supplementary Table S2). In general, this indicates that genetically predicted CD is causally associated with decreased levels of ALB and increased levels of ALP, and it also increases the risk of PBC and PSC.

In the replication analysis, 62 IVs (*F* = 70.36) were chosen to genetically predict UC, and 89 IVs (*F* = 76.98) were chosen to genetically predict CD. After removing all the outliers by MR-PRESSO, the MR estimates from specific methods of the causal effect of UC and CD on liver function traits and liver disease phenotypes are presented in a heatmap (Supplementary Figure S5). Significant MR analyses of UC on liver function traits and liver disease phenotypes are presented in Supplementary Table S3. Specifically, the causal effects of UC on ALB, liver iron content, and PSC were consistent with the previously mentioned analysis. Additionally, UC was associated with decreased levels of TC and LDL. While the findings indicated that genetically predicted UC was causally associated with an elevated risk of PBC (OR = 1.13, 95%CI 1.03 to 1.25, *p* = 0.006), the relationship between genetically predicted UC and PBC manifested inconsistent directional estimates across the IVW, MR-Egger, and WM methods. Significant MR analyses of CD on liver function traits and liver disease phenotypes are presented in Supplementary Table S4. Indeed, the causal effects of CD on ALP, PBC, and PSC were also consistent with the previously mentioned analysis. Furthermore, CD was associated with increased levels of AST and liver iron content, as well as decreased levels of APOB. Despite significant findings between CD and ALB (*β* = −0.023, 95%CI –0.038 to −0.008, *p* = 0.002), TC (*β* = −0.009, 95%CI –0.016 to −0.003, *p* = 0.001), as well as LDL (*β* = −0.005, 95%CI –0.01 to −0.0007, *p* = 0.022), inconsistent estimates were observed in their relationship. All the inconsistent estimates were viewed as suggestive evidence for a potential association.

## 4 Discussion

In the present study, we systematically evaluated the causal relationship between genetically predicted IBD and liver function traits, as well as liver disease phenotypes. The study yields robust conclusions, indicating that genetically predicted UC is causally associated with decreased levels of ALB and liver iron content. Additionally, it reveals that genetically predicted CD is causally associated with increased levels of ALP. Moreover, genetically predicted UC or CD increases the risk of PSC, and CD increases the risk of PBC. However, neither UC nor CD causally increases the risk of cholelithiasis and NAFLD.

The pathogenesis of liver injury in IBD remains unclear. The hepatic involvement seen in IBD patients can be classified into secondary diseases caused by IBD, co-occurring diseases alongside IBD, and hepatotoxicity induced by IBD medications. Accumulating evidence has shown that patients with IBD have liver function abnormalities and are susceptible to liver diseases (23–25). Our study found that CD is causally associated with increased levels of ALP. Elevation of ALP can be caused by liver damage or bone disorders. Indeed, elevated levels of ALP may occur in conjunction with various conditions among patients with IBD, including PSC, drug-induced liver injury (DILI), and bone metabolic disorders (26–28). PSC and bone metabolic disorders are well-documented extraintestinal manifestations of IBD (2). Meanwhile, individuals with IBD undergoing treatment with thiopurines, methotrexate, and biologics are at risk of developing DILI (27). The decreased levels of ALB caused by UC in the present study may be related to malnutrition induced by chronic inflammatory conditions or reduced albumin synthesis due to poor liver function. Additionally, we observed a decrease in liver iron content caused by UC. The liver acts as the central regulator of iron homeostasis, as it possesses the ability to detect and respond to iron stores by releasing the hormone hepcidin (29). However, while liver iron overload is a prevalent subject in various liver diseases, iron deficiency is a condition that can lead to a decrease in liver iron content. In the current study, the association between liver iron content and UC implies that liver iron content could be a consideration for patients with UC requiring an abdominal MRI scan.

The pathogenesis of PSC is still unclear, but the relationship between PSC and IBD suggests that intestinal factors may contribute to the development and progression of PSC (30). Clinical research has found that PSC can occur at any stage of IBD, and GWASs have identified multiple shared risk genetic loci between patients with IBD and PSC (14, 31). The pooled prevalence of PSC in patients with IBD was found to be 2.16%. Additionally, up to 85% of patients diagnosed with PSC also suffer from IBD (5, 6). The present study confirms the causal relationship between IBD and PSC. We also found that genetically predicted CD can increase the risk of PBC. Indeed, several sporadic cases detailing the association between PBC and IBD have been described (32). These findings further indicate that IBD could be considered a risk factor for the development of PSC and PBC. The underlying mechanisms of liver disorders in IBD may be closely related to the existence of the gut-liver axis. The gut–liver axis describes the mutual relationship between the gut, its microbiota, and the liver, which arises from the integration of signals originating from dietary, genetic, and environmental factors (33). The epithelial and gut vascular barriers are physical elements of the intestinal barrier that are disrupted in IBD (34, 35). The disruption of the intestinal barrier subsequently induces the translocation of bacteria or bacterial products, which then move through the portal vein directly to the liver and ultimately result in liver dysfunction.

In addition, several observational studies have reported that patients with CD have a higher prevalence of cholelithiasis compared to controls, but not in patients with UC (7, 36). The elevated occurrence of cholelithiasis among individuals with CD might be attributed to factors such as ileal resection and the use of total parenteral nutrition. However, in our study, CD and UC did not causally increase the risk of cholelithiasis. NAFLD is the leading cause of elevated liver enzymes in adults, and a meta-analysis based on observational studies revealed that IBD patients had a 2-fold higher risk of developing NAFLD compared to healthy individuals (4). Indeed, our study also demonstrated that genetically predicted CD, but not UC, is causally associated with an increased risk of NAFLD estimated by the IVW method (OR = 1.03, 95%CI 1.001 to 1.06, *p* = 0.04). Nonetheless, we could not draw a robust conclusion because of inconsistent directional estimates from the IVW, MR-Egger, and WM methods, and the LOO analyses revealed the presence of potentially influential instrumental variables in CD on NAFLD. On the other hand, the absence of an increase in percent liver fat in CD observed in this study also supports the estimate that CD does not increase the risk of NAFLD. Therefore, it suggests that we should cautiously interpret the relationship between IBD and cholelithiasis, or NAFLD, in observational studies.

There are several limitations to our study. First, the causal estimate from a Mendelian randomization investigation could not generally be interpreted directly as the expected impact of intervening on the exposure in practice. Mendelian randomization is used to gather evidence supporting a causal hypothesis, while further investigation is required to understand the underlying mechanisms of the hypothesis. For instance, the estimates that have a small effect size in the present study could not generally be interpreted directly as the expected impact. Second, the enrolled participants were European, so the estimates of IBD on liver function and disease in other ethnic populations remain unknown. Third, Mendelian randomization does not provide information on the timing of exposure and outcome. For instance, the duration of IBD is crucial for understanding various outcomes. Fourth, although the instrumental variables used in MR are robustly associated with IBD, they may not be entirely independent of potential confounding factors, such as specific IBD medications that may cause hepatotoxicity.

In conclusion, UC affects the levels of ALB and liver iron content, while CD impacts the levels of ALP. Additionally, both UC and CD increase the risk of PSC, with CD specifically increasing the risk of PBC. These significant results reinforce the findings of previous observational studies that suggest liver involvement as an extraintestinal manifestation of IBD, thus highlighting the existence of the gut–liver axis. Regular liver function monitoring is needed among patients with IBD.

## Data availability statement

The original contributions presented in the study are included in the article/Supplementary material, further inquiries can be directed to the corresponding authors.

## Ethics statement

Ethical approval was not required for the study involving humans in accordance with the local legislation and institutional requirements. Written informed consent to participate in this study was not required from the participants or the participants' legal guardians/next of kin in accordance with the national legislation and the institutional requirements.

## Author contributions

YS: Conceptualization, Formal analysis, Software, Writing – original draft. BY: Conceptualization, Data curation, Formal analysis, Writing – original draft. XL: Investigation, Methodology, Writing – review & editing. MX: Methodology, Resources, Writing – review & editing. CD: Data curation, Resources, Writing – review & editing. HW: Conceptualization, Methodology, Supervision, Validation, Visualization, Writing – review & editing.
