# Supplementary material for: Causal effects from inflammatory bowel disease on liver function and disease: a two-sample Mendelian randomization study
Source: Front Med (Lausanne). 2024 Jan 17;10:1320842. doi: 10.3389/fmed.2023.1320842 (PMC10827874; doi:10.3389/fmed.2023.1320842)
Supplement: Supplementary file 1 [file Data_Sheet_1.docx]

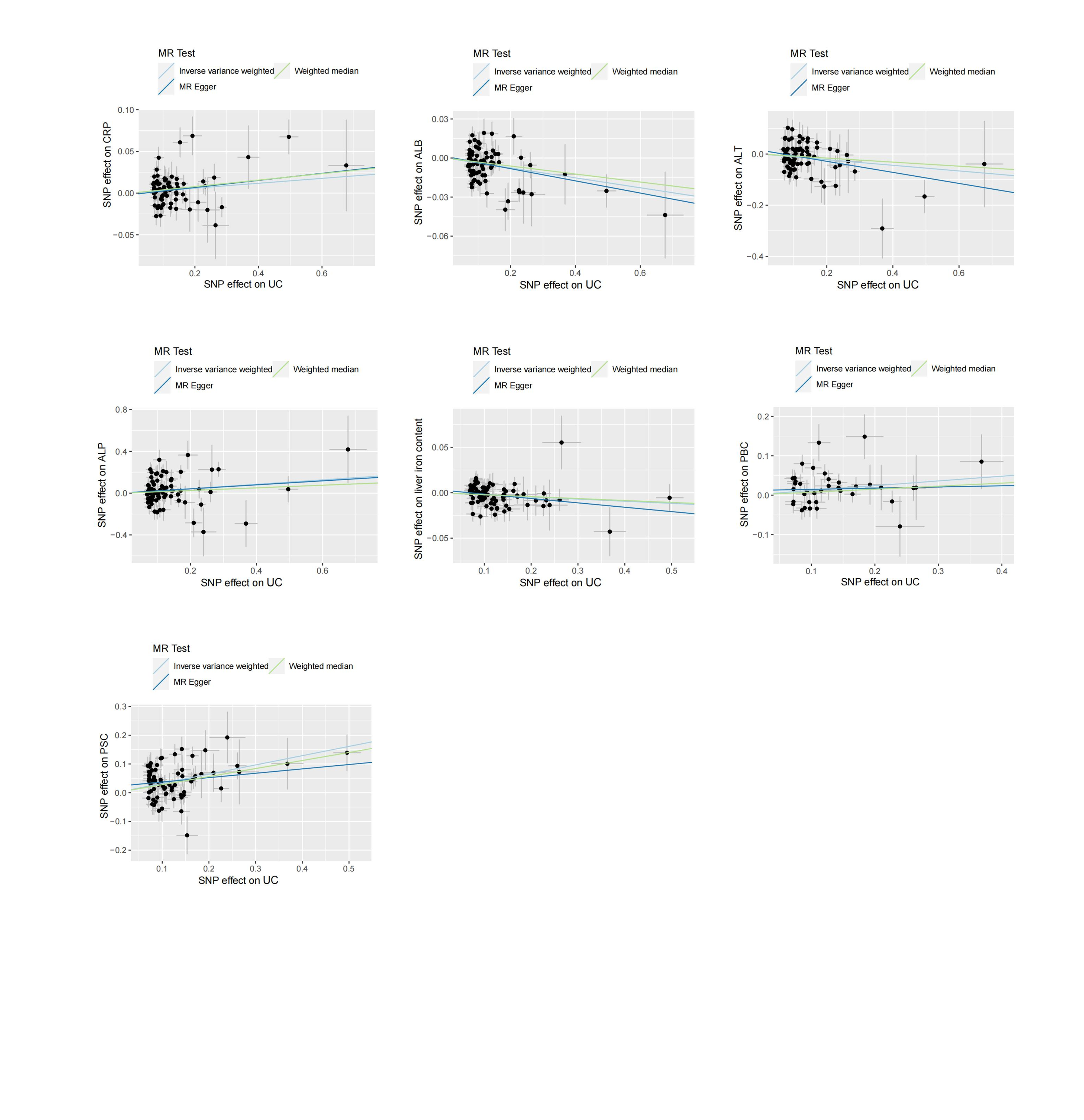


**Figure S1.** Scatter plots for significant Mendelian randomization association between UC and liver traits. CRP: C-reactive protein; ALB: albumin; ALT: alanine transaminase; ALP: alkaline phosphatase; PBC: primary biliary cholangitis; PSC: primary sclerosing cholangitis; UC: ulcerative colitis.


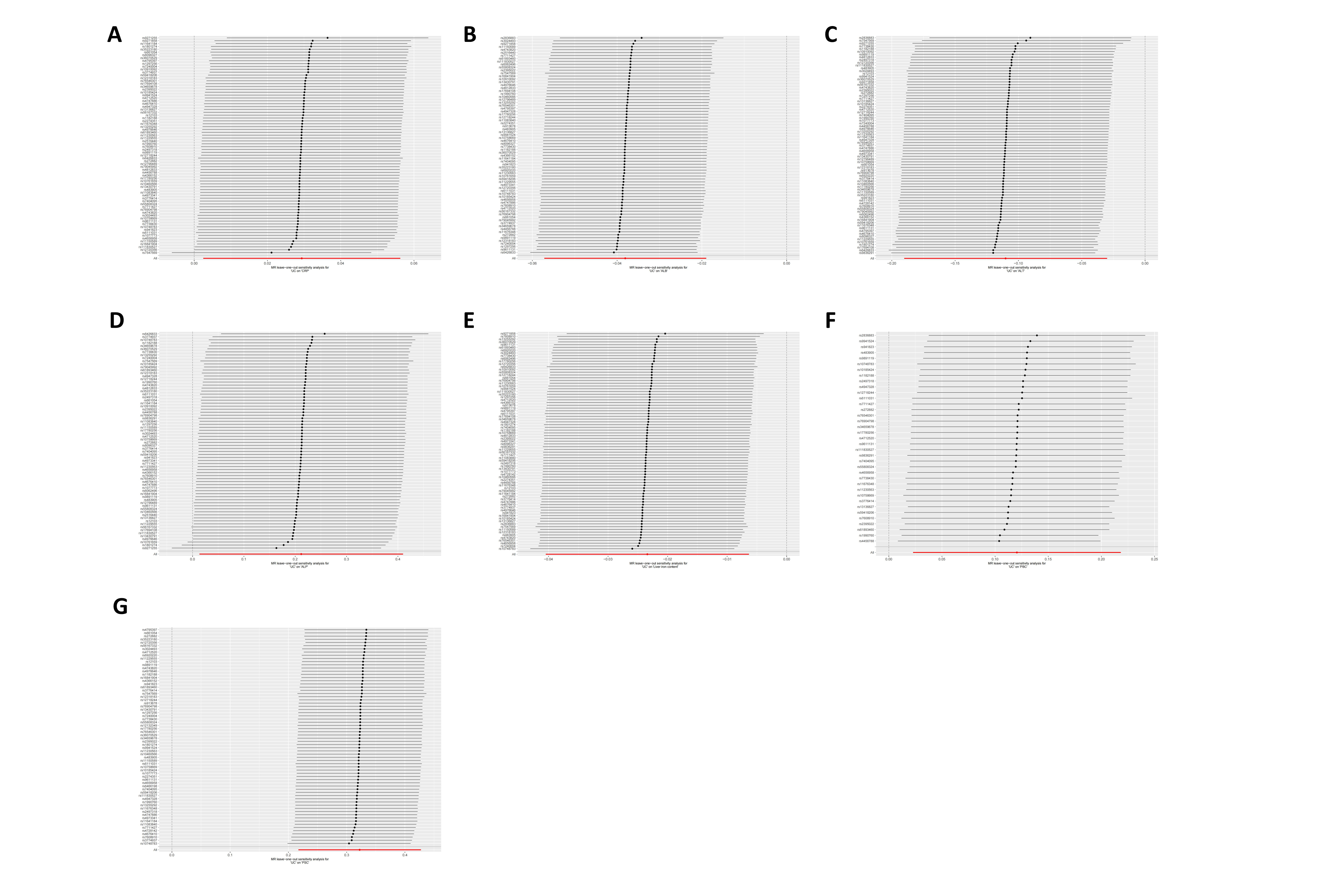


**Figure S2.** Leave-one-out sensitivity analysis for UC on liver traits. CRP: C-reactive protein; ALB: albumin; ALT: alanine transaminase; ALP: alkaline phosphatase; PBC: primary biliary cholangitis; PSC: primary sclerosing cholangitis; UC: ulcerative colitis.


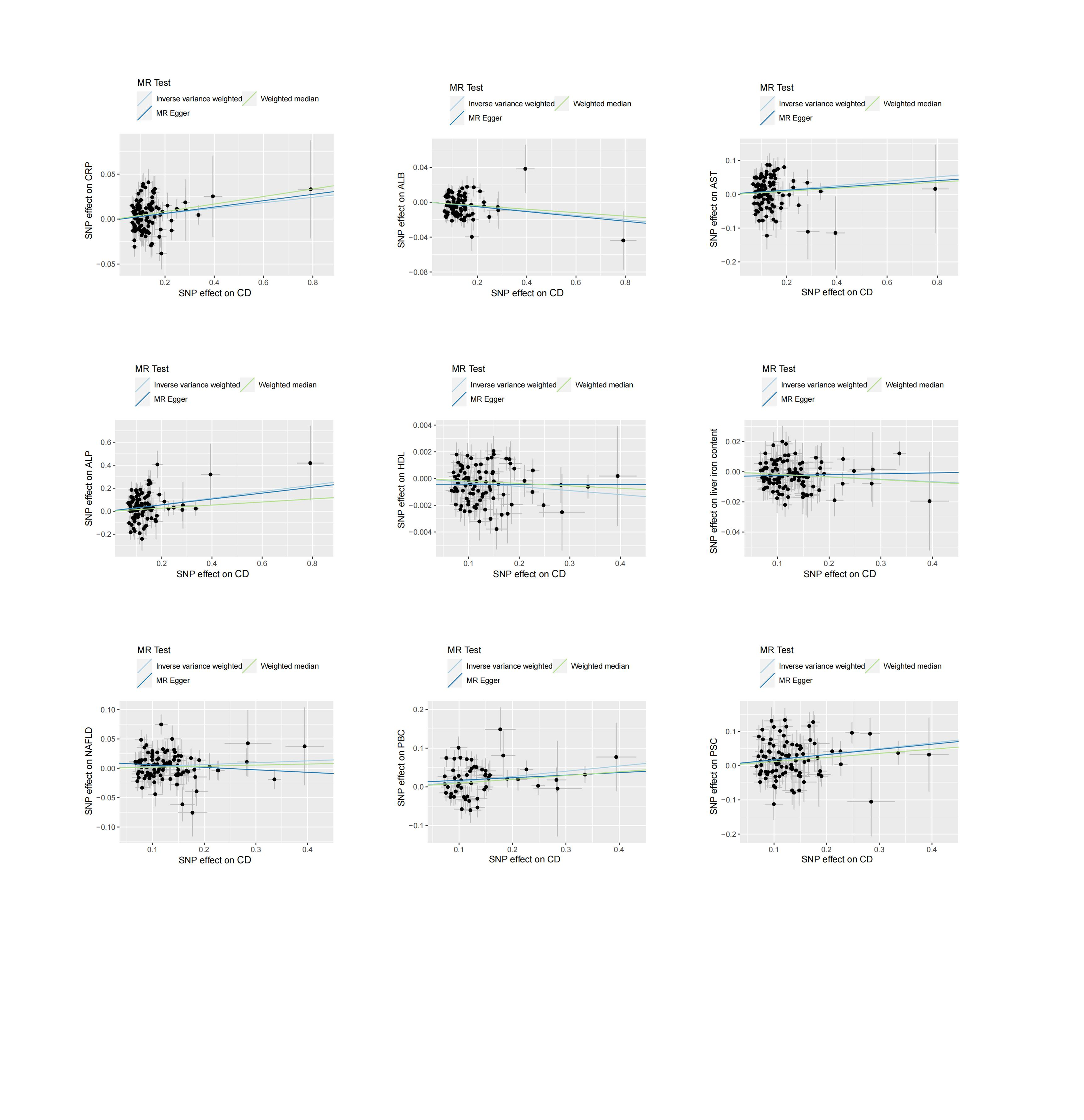


**Figure S3.** Scatter plots for significant Mendelian randomization association between CD and liver traits. CRP: C-reactive protein; ALB: albumin; AST: aspartate aminotransferase; ALP: alkaline phosphatase; HDL: high-density lipoprotein; NAFLD: nonalcoholic fatty liver disease; PBC: primary biliary cholangitis; PSC: primary sclerosing cholangitis; CD: Crohn’s disease.


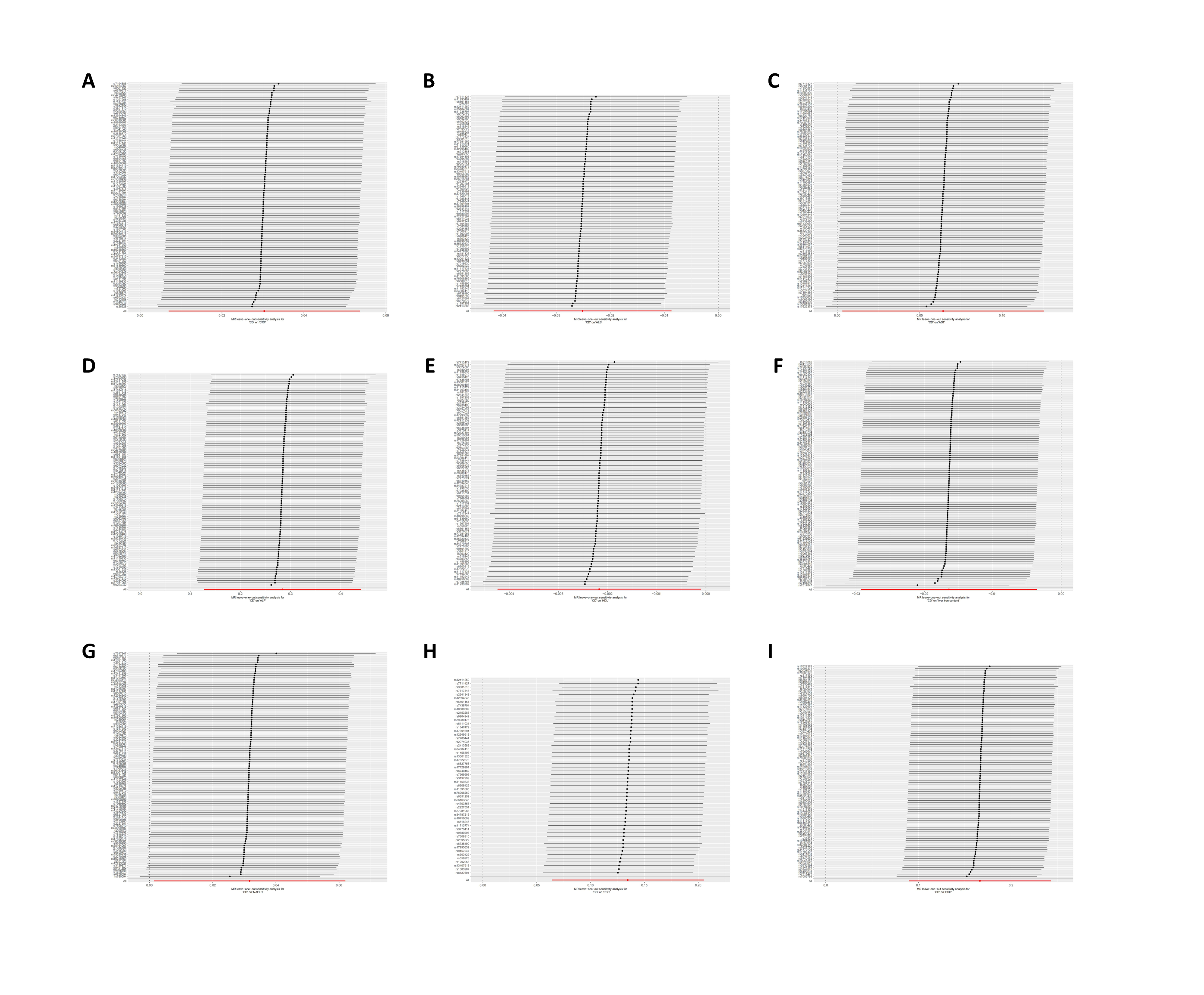


**Figure S4.** Leave-one-out sensitivity analysis for CD on liver traits. CRP: C-reactive protein; ALB: albumin; AST: aspartate aminotransferase; ALP: alkaline phosphatase; HDL: high-density lipoprotein; NAFLD: nonalcoholic fatty liver disease; PBC: primary biliary cholangitis; PSC: primary sclerosing cholangitis; CD: Crohn’s disease.


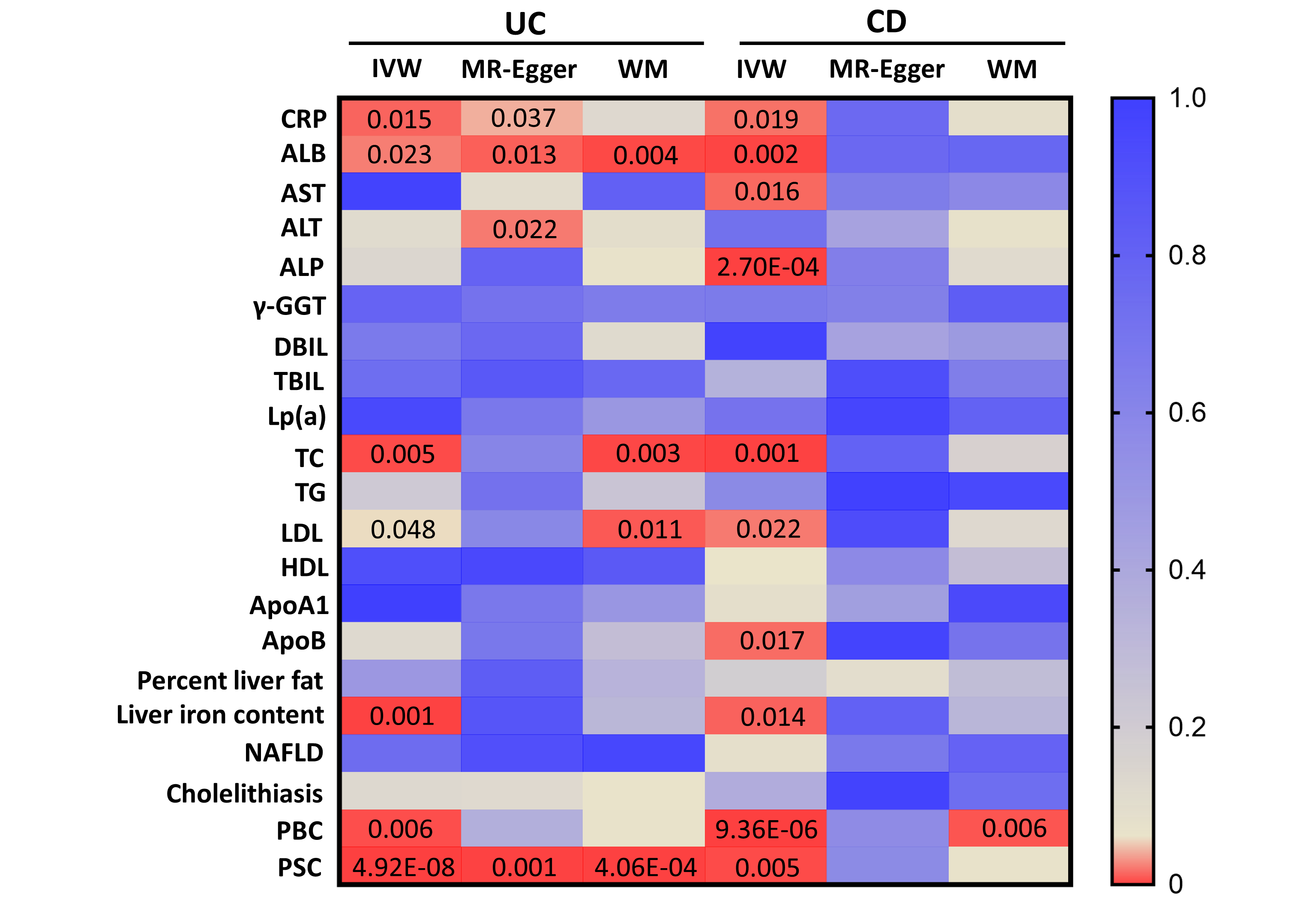


**Figure S5.** MR estimates from UC and CD on liver function traits and liver disease phenotypes. The color of each block represents the P-values of every MR analysis. CRP: C-reactive protein; ALB: albumin; AST: aspartate aminotransferase; ALT: alanine transaminase; ALP: alkaline phosphatase; γ-GGT: gamma-glutamyl Transferase; DBIL: direct bilirubin; TBIL: total bilirubin; Lp(a): lipoprotein (a); TC: total cholesterol; TG: triglyceride; LDL: low-density lipoprotein; HDL: high-density lipoprotein; ApoA1: apolipoprotein A1; ApoB: apolipoprotein B; NAFLD: nonalcoholic fatty liver disease; PBC: primary biliary cholangitis; PSC: primary sclerosing cholangitis; UC: ulcerative colitis; CD: Crohn’s disease; IVW: inverse variance weighted; WM: weighted median. *P* < 0.05 is set as significant.

**Table S1.** Steiger direction test from UC to liver traits.

| **Exposure** | **UC** | | | | | | |
| --- | --- | --- | --- | --- | --- | --- | --- |
| **Outcome** | **CRP** | **ALB** | **ALT** | **ALP** | **Liver iron content** | **PBC** | **PSC** |
| Direction | True | True | True | True | True | True | True |
| Steiger *P* | < 0.001 | < 0.001 | < 0.001 | < 0.001 | < 0.001 | < 0.001 | < 0.001 |

CRP: C-reactive protein; ALB: albumin; ALT: alanine transaminase; ALP: alkaline phosphatase; PBC: primary biliary cholangitis; PSC: primary sclerosing cholangitis; UC: ulcerative colitis.

**Table S2.** Steiger direction test from CD to liver traits.

| **Exposure** | **CD** | | | | | | | | |
| --- | --- | --- | --- | --- | --- | --- | --- | --- | --- |
| **Outcome** | **CRP** | **ALB** | **AST** | **ALP** | **HDL** | **Liver iron content** | **NAFLD** | **PBC** | **PSC** |
| Direction | True | True | True | True | True | True | True | True | True |
| Steiger *P* | < 0.001 | < 0.001 | < 0.001 | < 0.001 | < 0.001 | < 0.001 | < 0.001 | < 0.001 | < 0.001 |

CRP: C-reactive protein; ALB: albumin; AST: aspartate aminotransferase; ALP: alkaline phosphatase; HDL: high-density lipoprotein; NAFLD: nonalcoholic fatty liver disease; PBC: primary biliary cholangitis; PSC: primary sclerosing cholangitis; CD: Crohn’s disease.

**Table S3.** Significant MR analyses of UC on liver function traits and liver disease phenotypes.

| **Outcome** | **Method** | **Beta or OR_95CI** | **P** | **Q** | **Qdf** | **Intercept_P** |
| --- | --- | --- | --- | --- | --- | --- |
| CRP | IVW | 0.033 (0.006, 0.06) | 0.015 | 29 | 35 |  |
|  | MR Egger | 0.096 (0.009, 0.184) | 0.037 | 27 | 34 | 0.144 |
|  | Weighted Median | 0.033 (–0.007, 0.073) | 0.109 |  |  |  |
| ALB | IVW | –0.022 (–0.041, –0.003) | 0.023 | 69 | 42 |  |
|  | MR Egger | –0.082 (–0.144, –0.019) | 0.013 | 63 | 41 | 0.056 |
|  | Weighted Median | –0.032 (–0.055, –0.01) | 0.004 |  |  |  |
| TC | IVW | –0.012 (–0.02, –0.003) | 0.005 | 93 | 46 |  |
|  | MR Egger | –0.009 (–0.037, 0.018) | 0.519 | 93 | 45 | 0.834 |
|  | Weighted Median | –0.014 (–0.024, –0.004) | 0.003 |  |  |  |
| LDL | IVW | –0.006 (–0.012, –0.00003) | 0.048 | 88 | 46 |  |
|  | MR Egger | –0.007 (–0.028, 0.013) | 0.505 | 88 | 45 | 0.942 |
|  | Weighted Median | –0.009 (–0.016, –0.002) | 0.011 |  |  |  |
| Liver iron content | IVW | –0.027 (–0.044, –0.01) | 0.001 | 53 | 50 |  |
|  | MR Egger | –0.006 (–0.064, 0.05) | 0.824 | 52 | 49 | 0.465 |
|  | Weighted Median | –0.013 (–0.037, 0.01) | 0.26 |  |  |  |
| PSC | IVW | 1.35 (1.21, 1.51) | 4.92e-08 | 81 | 37 | 0.179 |
|  | MR Egger | 1.61 (1.23, 2.11) | 0.001 | 77 | 36 |  |
|  | Weighted Median | 1.27 (1.11, 1.46) | 4.06e-04 |  |  |  |

**Table S4.** Significant MR analyses of CD on liver function traits and liver disease phenotypes.

| **Outcome** | **Method** | **Beta or OR_95CI** | **P** | **Q** | **Qdf** | **Intercept_P** |
| --- | --- | --- | --- | --- | --- | --- |
| CRP | IVW | 0.038 (0.006, 0.07) | 0.019 | 296 | 80 |  |
|  | MR Egger | 0.017 (–0.068, 0.104) | 0.684 | 296 | 79 | 0.624 |
|  | Weighted Median | 0.026 (–0.003, 0.055) | 0.078 |  |  |  |
| AST | IVW | 0.069 (0.012, 0.125) | 0.016 | 151 | 74 |  |
|  | MR Egger | 0.041 (–0.1, 0.184) | 0.569 | 150 | 73 | 0.681 |
|  | Weighted Median | 0.022 (–0.043, 0.088) | 0.497 |  |  |  |
| ALP | IVW | 0.268 (0.124, 0.413) | 2.70e-04 | 135 | 68 |  |
|  | MR Egger | 0.109 (–0.253, 0.472) | 0.555 | 133 | 67 | 0.351 |
|  | Weighted Median | 0.145 (–0.029, 0.32) | 0.102 |  |  |  |
| APOB | IVW | –0.001 (–0.002, –0.0002) | 0.017 | 137 | 71 |  |
|  | MR Egger | –0.00006 (–0.003, 0.003) | 0.972 | 136 | 70 | 0.371 |
|  | Weighted Median | –0.0003 (–0.001, 0.001) | 0.622 |  |  |  |
| Liver iron content | IVW | –0.015 (–0.027, –0.002) | 0.014 | 65 | 76 |  |
|  | MR Egger | –0.005 (–0.037, 0.027) | 0.745 | 64 | 75 | 0.521 |
|  | Weighted Median | –0.01 (–0.029, 0.008) | 0.266 |  |  |  |
| PBC | IVW | 1.13 (1.07, 1.20) | 9.36e-06 | 86 | 44 |  |
|  | MR Egger | 1.04 (0.91, 1.19) | 0.483 | 82 | 43 | 0.185 |
|  | Weighted Median | 1.09 (1.02, 1.17) | 0.006 |  |  |  |
| PSC | IVW | 1.12 (1.03, 1.22) | 0.005 | 122 | 57 |  |
|  | MR Egger | 1.09 (0.84, 1.41) | 0.489 | 121 | 56 | 0.812 |
|  | Weighted Median | 1.09 (0.99, 1.21) | 0.06 |  |  |  |
